# Supplementary material for: Targeted gene transfer into developmentally defined cell populations of the primate brain
Source: bioRxiv. 2025 Apr 17:2025.04.11.648413. Preprint. [Version 1] doi: 10.1101/2025.04.11.648413 (PMC12190174; doi:10.1101/2025.04.11.648413)
Supplement: Supplement 1 [file media-1.pdf]

Supplementary Table 1: Viral constructs used in this study

Serotype	AAV Vector	Packaged by	Unique ID	Addgene Plasmid	Titer range (VG/mL)	Gift from	Used for
AAV2	pAAV-CAG-GFP	Addgene	37825-AAV2	37825	5.30E+12 - 1.5E+13	Edward Boyden	Serotype evaluation
AAV8	pAAV-nEF-Con/Foff 2.0-ChRmine-oScarlet	Addgene	137161-AAV8	137161	2.10E+13	Karl Deisseroth & INTRSECT 2.0 Project	Cre/Lox recombination-based intersectional strategy
AAV9	pAAV-CAG-GFP	Addgene	37825-AAV9	37825	2.60E+13	Edward Boyden	Gestation timing and serotype evaluation
AAV9	pAAV-CAG-tdTomato (codon diversified)	Addgene	59462-AAV9	59462	2.3E+13 - 2.5E+13	Edward Boyden	Gestation timing evaluation
AAV9	pAAV-NestinTK-EGFP-iCre	NIDA GEVVC	AAV916	228443	4.29E+12	(in house)	Cre/Lox recombination-based intersectional strategy
AAV9	pAAV-rActin-EGFP-donor	NIDA GEVVC	AAV942	228444	1.96E+13	(in house)	CRISPR/Cas9-mediated genome editing
AAV9	AAV9-EFS-SpCas9	NIDA GEVVC	AAV949	104588	1.40E+13	(in house)	CRISPR/Cas9-mediated genome editing

Supplementary Table 2: Plasmids used in this study

Plasmid	Source	Catalog number	Addgene Plasmid	Gift from	Used for
pNestin-EGFP	Addgene	38777	38777	Wei Cui	Template for cloning, pOTTC2234
pAAV-HDR-mEGFP-Actin	Addgene	119870	119870	Ryohei Yasuda	Template for cloning, pOTTC2360
pAAV-EFS-SpCas9	Addgene	104588	104588	Ryohei Yasuda	Packaged as vector in top section
pAAV CMV-IE eGFP-2A-iCre	NIDA GEVVC	pOTTC1031	n/a	NIDA GEVVC	Template for cloning, pOTTC2234
pAAV JeT ConFon HA-hM4D(Gi)-mCherry TK65pA	NIDA GEVVC	pOTTC2210	n/a	NIDA GEVVC	Template for cloning, pOTTC2234
pAAV Nestin GFP-iCre	NIDA GEVVC	pOTTC2234	228443	this study	Packaged as vector in top section
pAAV rActin EGFP donor	NIDA GEVVC	pOTTC2360	228444	this study	Packaged as vector in top section
pHelper	PENN Vector Core	n/a	n/a	PENN Vector Core, Perelman School of Medicine, University of Pennsylvania	AAV packaging plasmids, in trans
pAAV 2/9	PENN Vector Core	n/a	n/a	PENN Vector Core, Perelman School of Medicine, University of Pennsylvania	AAV packaging plasmids, in trans

Supplementary Table 3: Setup and procedure tools

Anesthesia machine and monitoring systems

Product	***Name***	***Part #***	***Brand***	***Reusable***	***Custom-made***
Anesthesia machine	RC2 Rodent Circuit Controller	922100	VetEquip	Y	N
Induction box (rat and neonatal marmosets)	Induction Chamber, 7 liter	941488	VetEquip	Y	N
Conductive gas supply hose	Color coded, nylon-reinforced, 1/4" ID, conductive gas supply hose	931503	VetEquip	Y	N
Rat nose cone	12mm Nosecone and 14mm Nosecone	921612 and 921614	VetEquip	Y	N
Marmoset nose cone	Anesthesia silicone mask, reusable, size 00, premature baby	MP02900	Drager	Y	N
Infrared heater	NORMOTHERM™ INFRARED HEATER	n/a	Britz & Company	Y	N
Rat monitoring system	SomnoSuite® Low-Flow Anesthesia System	SS-01	Kent Scientific Corporation	Y	N
Marmoset monitoring system	IntelliVue MX500 Patient Monitor	866064	Philips Medizin Systeme Böblingen GmbH	Y	N
Marmoset EKG leads	Micro NeoLead, AAMI radio lead	989803183141	Philips Medical Systems Hsg	Y	N
SpO2 Sensor	Nasal Alar SpO2 Sensor	989803205381	Philips Medical Systems Hsg	Y	N
Blood pressure cuff	NIBP Cuffs (Neonatal single-patient cuff size #1)	M1866B	Philips Medical Systems Hsg	Y	N

Electrosurgery unit

Product	***Name***	***Part #***	***Brand***	***Reusable***	***Custom-made***
Electrosurgery Unit	Symmetry Pro-120 Multi-Pupose Electrosurgical Generator	A1250S	Bovie	Y	N
Electrosurgical Pencil	Push Button Electrosurgical Pencil 50/Box	ESP1	Bovie	Y	N
Reusable Grounding Cable	Reusable Grounding Cable	A1252C	Bovie	Y	N
Return Electrodes	Disposable Split Adult Return Electrodes	ESRE-1	Bovie	N	N
Sterile US gel	Aquasonic 100 - Sterile Single Use - Overwrapped Foil Pouches 48 per box	PLI 01-01	Aquasonic	N	N

Guides and needles

Product	***Name***	***Part #***	***Brand***	***Reusable***	***Custom-made***
Guide tubes	23 gauge, Hubless Needle, custom length (2 in), point style 4, 12 DEG, 6/PK	22023-01	Hamilton Company	Y	Y
	24 gauge, Hubless Needle, custom length (2 in), point style 4, 12 DEG, 6/PK	22024-01	Hamilton Company		
Needles	33 gauge, Small Hub RN Needle, custom length (2.5 in), point style 4, 12 DEG, 6/PK	7803-05	Hamilton Company	Y	Y
	31 gauge, Small Hub RN Needle, custom length (2.5 in), point style 4, 12 DEG, 6/PK	7803-03	Hamilton Company		
Syringe	5 µL, Model 75 RN Syringe, Needle Sold Separately	7634-01	Hamilton Company	Y	N
	10 µL, Model 701 RN Syringe, Needle Sold Separately	7635-01	Hamilton Company		
	100 µL, Model 710 RN Syringe, Needle Sold Separately	7638-01	Hamilton Company		
Heat shrink tubing	Palladium™ "Pebax™" Heat Shrink Tubing	PBST2-040-40-004C	Component Supply	N	N
Heat gun	1500 Watt 10 Amp 12 Temperature Heat Gun	69343	Harborfreight	Y	N

Injection setup

Product	***Name***	***Part #***	***Brand***	***Reusable***	***Custom-made***
18.70 mm A/P Bar	Model 1400 series	n/a	KOPF	Y	N
A.P. Slide Attachment	Model 1261 A/P Slide Attachment	n/a	KOPF	Y	N
Rotation Adapter	Model 1460-G Rotation Adapter	n/a	KOPF	Y	N
Travel Vertical Translation Stage	VAP4/M - 101.6 mm Travel Vertical Translation Stage, M4 and M6 Taps	VAP4/M	KOPF	Y	N
Stereotaxic arm and micro manipulator with fine adjustm	Model 1460-61 electrode Carrier with Fine Adjustment A/P Slide Assembly	1460-61	KOPF	Y	N
Single Axis Translation Stage	LT1/M - Single Axis Translation Stage, 50 mm Travel, Metric	LT1/M	THORLABS	Y	N
Cube Geared Head	Arca-Swiss C1 Cube Geared Head with Arca Classic Quick	8501303.1	Arca-Swiss	Y	N
Aluminum Breadboard	Aluminum Breadboard, 250 mm x 300 mm x 12.7 mm, M6 Taps	MB2530/M	THORLABS	Y	N
Animal cradle	n/a	n/a	NIMH/SI	Y	Y
Stereotaxic arm base holder	n/a	n/a	NIMH/SI	Y	Y
Ultrasound probe holder	n/a	n/a	NIMH/SI	Y	Y
Costum plates	n/a	n/a	NIMH/SI	Y	Y
Stainless steel extended spring clip	n/a	n/a		Y	N
Loc-Line® 0.5"	n/a	n/a		Y	N

Ultrasound machine and transducers

Product	***Name***	***Part #***	***Brand***	***Reusable***	***Custom-made***
US machine	Vevo MD Imaging System	51475	Fujifilm Sonosite, Inc.	Y	N
US transducers	UHF70 Transducer	51416	Fujifilm Sonosite, Inc.	Y	N
	UHF48 Transducer	51415	Fujifilm Sonosite, Inc.		
	UHF22 Transducer	51414	Fujifilm Sonosite, Inc.		

Other

Product	***Name***	***Part #***	***Brand***	***Reusable***	***Custom-made***
Isofluran	Multiple sources	n/a	n/a	N	N

Hair removal creme	Nair Body Cream	n/a	Church & Dwight Co., Inc.	N	N
Eye lubricant	Multiple sources	n/a	n/a	N	N
Ethanol wipes	Multiple sources	n/a	n/a	N	N
Povidone iodine	Betadine Surgical Scrub (7.5% povidone-iodine) Antiseptic Non-Sterile Scrub	67618-154-16	Purdue Products LP	N	N
Lidocaine	Multiple sources	n/a	n/a	N	N
2x2 Gauze	Pivetal Non-Woven Gauze Sponge 2in. x 2in. (Gauze)	21295051	Patterson Veterinary	N	N
4x4 Gauze	Pivetal Non-Woven Gauze Sponge 4in. x 4in. (Gauze)	21295051	Patterson Veterinary	N	N
Hair trimmer	Bravmini+ Purple	41590-0438	WAHL	Y	N
Hair trimmer blade	Bravmini+ Designer Blade	41590-7840	WAHL	Y	N
Tong depressors	Sterile Regular Tongue Depressors (Tongue Depressors)	25-705	Puritan Medical Products	N	N
Cotton Swab	Sterile Cotton Tipped Applicators (Sterile Applicators)	56800	AMD-Ritmed	N	N
Tape	Transpore Surgical Tape (tape)	1527-1	3M	N	N

Experimental models: organisms/strains	**Strain**	***Supplier***
Rattus norvegicus	CD® IGS (Sprague Dawley) Rats	Charles River
Callithrix jacchus		Worldwide Primates Inc. and in-house breeding colony

Supplementary Table 4: Histological processing

Reagent	Source	Identifier
Chemicals		
Paraformaldehyde	Multiple sources	Cas# 30525-89-4
Glycerol	Sigma	Cat# G5516, Cas# 56-81-5
Tissue-Tek® O.C.T Compound	Electron Microscopy Science (Sakura Finetek)	Cat# 62550-12
Fluoromount™ Aqueous Mounting Medium	Sigma	Cat# F4680
4',6-diamidino-2-phenylindole (DAPI)	Thermo Fisher Scientific	Cat# 62248
Ethyl alcohol, Pure 200 proof.	Multiple brands	Cas# 64-17-5
Sodium chloride	Multiple brands	Cas# 7647-14-5
Sodium dodecyl sulfate	Sigma	Cat# 75746, Cas# 151-21-3
Boric acid	Thermo Scientific Chemicals	Cat# 12680, Cas# 10043-35-3
Sodium sulfite	Sigma-Aldrich	Cat# S0505, Cas# 7757-83-7
Antipyrine Crystalline	Sigma-Aldrich	Cat# A5882, Cas# 60-80-0
Nicotinamide	Sigma	Cat# 72340, Cas# 98-92-0
N-butyldiethanolamine	Sigma	Cat# 471240, Cas# 102-79-4

Primary antibodies		***Identifier***	***Working dilution***
Chicken anti-Green Fluorescent Protein Antibody	Aves Labs	Cat# GFP-1020, RRID:AB_100002	1:5000
Guinea pig anti-NeuN Antibody	Millipore	Cat# ABN90P, RRID:AB_2341095	1:5000
Mouse anti-NeuN Antibody	Millipore	Cat# MAB377, RRID:AB_2298772	1:5000
Mouse anti-parvalbumin Antibody	Swant	Cat# 235, RRID:AB_10000343	1:2000

Secondary antibodies	***Brand***	***Identifier***	***Working dilution***
Goat anti-Guinea Pig IgG (H+L) Highly Cross-Adsorbed Secondary Antibody, Alexa Fluor™ 555	Thermo Fisher Scientific	Cat# A-21435, RRID:AB_2535856	1:1000
Goat anti-Chicken IgY (H+L) Secondary Antibody, Alexa Fluor 488	Thermo Fisher Scientific	Cat# A-11039, RRID:AB_2534096	1:1000
Goat anti-Mouse IgG (H+L) Highly Cross-Adsorbed Secondary Antibody, Alexa Fluor™ 647	Thermo Fisher Scientific	Cat# A-21236, RRID:AB_2535805	1:1000
